# Age-dependent survival rate of the colonial Little Tern (*Sternula albifrons*)

**DOI:** 10.1371/journal.pone.0226819

**Published:** 2019-12-31

**Authors:** Inbal Schekler, Yosef Kiat, Roi Dor

**Affiliations:** 1 School of Zoology, Faculty of Life Sciences, Tel Aviv University, Tel Aviv, Israel; 2 Department of Evolutionary and Environmental Biology, University of Haifa, Haifa, Israel; 3 Israel Ornithological Center, Society for the Protection of Nature in Israel, Tel Aviv, Israel; University College Dublin, IRELAND

## Abstract

Many ground-nesting bird species are suffering from habitat loss and population decline. Data on population ecology and demography in colonies of threatened species are thus essential for designing effective conservation protocols. Here, we used extensive ringing and observation data to estimate directly, for the first time, the survival rate of juvenile and adult Little Tern (*Sternula albifrons*), as well as testing for a possible effect of age on probability of survival. We estimated adult annual survival rate to be 0.77, and juvenile (first year) survival to be 0.49 with a possible linear decrease in the survival rate of the juveniles that ranged from 0.681 to 0.327. We found no evidence that survival was age-dependent among the early age classes after the first year. We discuss these findings in light of survival estimates for other species, and their implications for the Little Tern conservation.

## Introduction

Populations of many ground-nesting bird species have been decreasing in recent years, mainly due to habitat loss and fragmentation [1,2]. Conservation of seabird colonies has become more complicated with the increase in threats posed by human disturbance, such as the development of coastal sites, increased pollution, habitat degradation, and the presence of invasive predators [3]. Effective conservation actions necessitate input from scientific knowledge on many aspects of the birds' biology, including population dynamics and demography, behavior, life history, and ecology [3–5]. Among the life-history characters of birds, reliable data on adult survival, and especially juvenile survival and the differences between survival at different age cohorts, are scarce and the data are difficult to estimate [1]. However, such data, and the temporal trends in these parameters, are important for an understanding of the population dynamics [2,6]. Empirical data on juvenile survival can improve our assessment of population productivity and viability, enable identification of conservation problems, and enable better assessment of the breeding habitat quality [7].

Studies of long-lived birds have suggested a pattern of successive increase in the average survival probability through the first years of an individual's life [3,8–11]. This pattern can be explained by the "selection hypothesis" or "selective disappearance" [4], where natural selection progressively eliminates low-quality individuals. Thus, the proportion of high-quality individuals should increase through age classes, with a consequent increase in survival with age [12]. Another possible explanation is that of the "average ontogenetic development", according to which there is a positive effect of experience and improvement in skills on the survival and, therefore, here too survival is expected to increase with age [4,13].

The Little Tern (*Sternula albifrons*) is a migratory seabird that breeds in colonies along sandy seashores and river banks [3]. Little Tern populations have been declining over the last two centuries [6,14], mainly due to the loss of natural breeding habitats. This tern has narrow habitat requirements and nests on open sandy places, with little or no vegetation, at low elevation, mostly along the coast but also in estuaries and on large islands [3]. In response to habitat loss over the last decades it has also begun to breed in alternative sites such as salinas (man-made salt-pans) [15] and modified rooftops [16]. While seabirds generally display high fidelity to breeding sites, terns can show high rates of dispersal and can rapidly colonize new sites in response to changes in habitat quality [17,18]. Despite the importance of data on survival estimates for the conservation of this species, there is very little published information due to the relatively low recovery rates of the Little Tern [3]. In Israel, the Little Tern has suffered from habitat loss and its population has declined by over 80% over the last three decades [19]. It has thus been listed as 'Endangered' since 2017 [20,21]. Recently, Little Terns in Israel have been mainly restricted to breeding at one site on an artificial island inside man-made salt evaporation ponds, as well as at a smaller nearby reservoir. Both sites demand constant management to ensure their continued use as breeding sites. However, despite the protection efforts there are no data available regarding the terns’ survival in these colonies.

The aim of the present study was to estimate for the first time directly adult and juvenile survival rates of Little Terns, and to address the effect of age on survival among the younger age classes. We analyzed multiple-year information of 436 known-age Little Terns using capture-recapture models, which estimate probability rate of recapture and survival rate separately.

## Methods

### Study site and breeding history

The main and almost exclusive breeding site of the Little Tern in Israel is an artificial island (ca. 60x50 m), located on the salt ponds of the salt factory in Atlit (hereafter 'Atlit'; 32.69°N, 34.93°E; [22]). The island is bare of vegetation except for two small bushes, and is also the main breeding site for the Common Tern (*Sterna hirundo*) in Israel. The colony of Little Terns there consisted of 250 pairs in the early 1990s, but in 2010 there were only a few dozen pairs still breeding at Atlit, due to an increase in predation and the flooding of nests [21]. In 2011 the artificial island was fenced in order to exclude land predators, and since then the number of breeding pairs had grown to about 250 pairs in 2015–2016.

### Trapping, marking and observations

We trapped terns using mist nets (126 m of mist nets; 0.5–3.5 m above water level) in the salt ponds at Atlit during the breeding season (April to August, 2011–201**8**). Nets were opened at sunset and closed between midnight and sunrise, according to trapping success. The terns were attracted to the nets by playbacks of tern calls and by tern dummies. We did not trap terns between late May and late June, since they incubate during this period and rarely fly. During the spring (April to May), we trapped terns every 5 days, and during the summer (late June to August), once a week. Each tern was marked with steel and plastic coded rings that enabled individual identification from a distance. Terns were released immediately after the ringing process at the site. We assessed each tern’s age as either 'juvenile—first year' (hereafter juvenile) or 'more than first year' (hereafter adult) according to its plumage. In total, 1,182 Little Terns were ringed, 5**24** of which were trapped as juveniles.

Observations of the ringed individuals were collected from birdwatchers using binoculars and telescopes from April to August. Since 2014 more intensive observations were carried out as part of the research project, at least twice a week, and were performed, in addition to the binoculars and spotting scopes, using an online camera located on the island and controlled through a computer and from a hide located on the bank of the pond.

### Survival and recapture analyses

We used data derived from 436 Little Tern individuals ringed as juveniles from 2011 to 2016 at one of the breeding sites in Israel (Atlit), in order to calculate the survival rate of adults and juveniles. Juvenile survival was estimated only until 2016 because a large percentage of the juveniles return to the breeding sites only during their third year [3]. There is an additional breeding site in Israel located 10 km south of Atlit, where the terns breed later during the season, not every year, and in smaller numbers (30–80). At this site we ringed only two juveniles among the years, and to avoid site effects, we exclude this record from the analysis. We used all the resightings data (of individuals ringed as juveniles) we had, which include the years 2011–2018, and mainly data from Atlit but also from the surrounding areas (with more than 99% of the data from within 15 km from Atlit and 81.7% of the data from observations, and not recaptures). The next closest colony of Little Tern is located about 230 km south of Atlit and we have no resighting data from it (nor any other breeding site outside of Israel), and therefore we have treated this population as a closed one. Little Tern survival (ф) and recapture (p) probabilities were estimated over annual intervals according to the Cormack-Jolly-Seber (CJS) models [23,24] using the MARK program [25]. The parameters of ф in CJS models represents the probability of an individual surviving and not permanently emigrating. The parameter of p in these models represents the probability of resighting an individual present in the study area, and also incorporates temporary emigration [26]. We chose only the individuals ringed as juveniles (436 individuals) in order to use only individuals for which we knew their exact age and to avoid underestimation of survival by including migratiory birds (that only pass through Atlit and therefore have lower chance of resighting). Age and time-based models were parameterized to contain different structures. Survival and resightings were examined for differences between each age of the bird (first year until 4 year), between the first year (juveniles) and after the first year (adult), and between the first year and the second year, to more than the second year. Sets of candidate models were chosen prior to data analysis, based on our knowledge of the Little Tern biology (Burnham & Anderson 1998; S1 Table). Model Notation for ф and p: '(.)' = constant over time, '(t)' = annual variation, '(T)' = linear time trend, (t/t) = including interaction between age and time, (+t) = additive effect of time (without interaction), a2–2 age classes (juvenile and adult), a3–3 age classes (juvenile, second year and after second year), a**4**–**4** age classes (juvenile, second year, third year, and after third year). Symbols separating age classes indicate whether they were combined (=) or estimated separately. For example, the model ф (a2 –t/.) represents two age classes for survival (a2): juvenile (first) age class time-dependent, and adult age class constant over time (t/.)

The general, or global, model ф (**4**a –t/t/t/t), p (4a –t/t/t/t) includes the effect of age (first year, second year, third year and after the third year), and time, on survival and resightings. The survival probability was assumed to vary according to age, with increasing survival during the first years of a bird's life. The resightings probability was assumed to vary according to time as a result of the different efforts devoted to reading ringed individuals in the field across the years. In addition, due to decrease in the probability to stay in Africa with age [3], resighting probability was assumed to vary with age as well.

The general model was tested with the program UCARE V2.3.4 [27] and the median c-hat procedure available in "Program MARK". Models in each candidate set were first ranked by second-order AICc differences (ΔAICc) [28]. Relative likelihood of each model in a candidate set was then estimated with AICc Weights. Parameter estimates were obtained from the model with the lowest AICc score.

### Ethics statement

All research procedures, including capturing, ringing, observations, and modifications comply with the current laws of Israel, and were carried out under permits from the Israel Nature and Parks Authority (INPA) (permit approvals #2014/ 40318, #2015/ 40747, #2016/ 41306, #2017/41686, #2018/41939). Tel Aviv University Animal Care and Use Committee granted a formal waiver of ethics approval.

## Results

### Survival, and recapture rate

The general model (ф (a4—t/t/t/t) p (a4—t/t/t/t)) fits the data well both by U-CARE V2.3.4 (χ^2^_17_ = 18.595, *P* = 0.352) and by the median c-hat procedure (c-hat = 0.977, SE = 0.0028).

**Fig 1:**
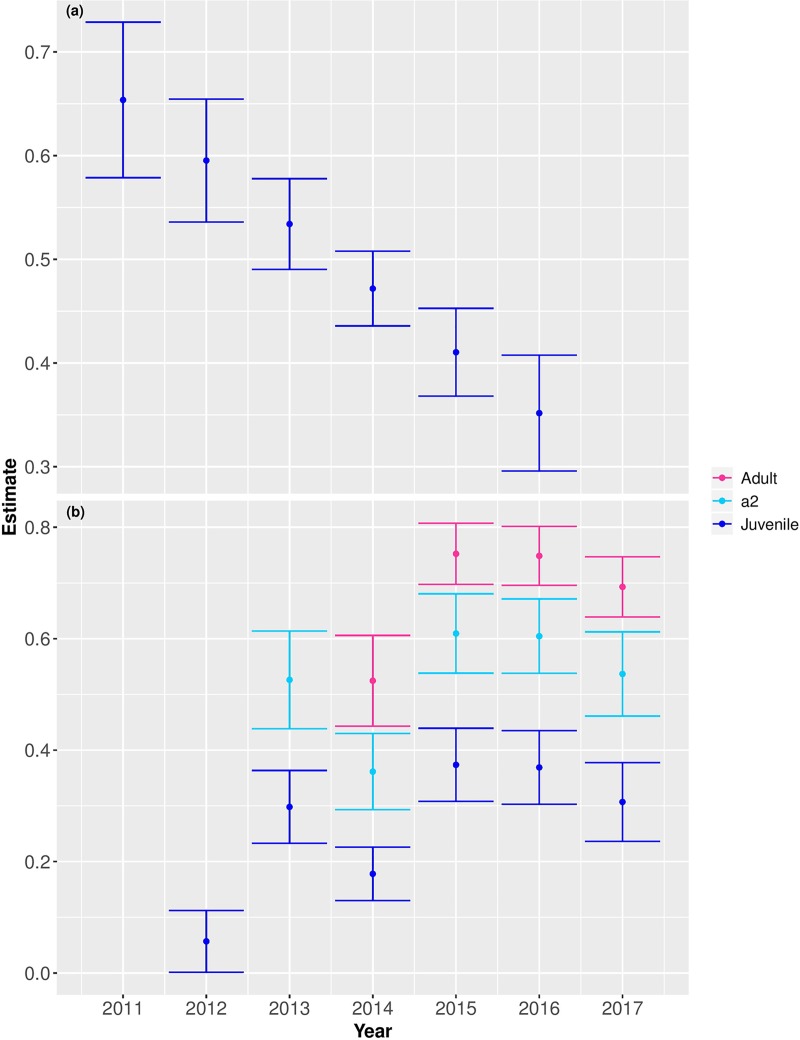
Estimates of (a) apparent survival (± SE) and (b) resigthing (± SE) from the top models for juvenile (in blue), a2 (second year; in light blue) and adult (in red) Little Terns from 2011–2017. Juvenile survival was estimated only until 2016 because a large percentage of individuals return to the breeding sites only in their third year.

The best resighting probability models included different resighting rates for 3 or 4 age classes and indicated the additive effect of annual variation (t; Table 1, two top models, Fig 1B). These models (p(a4 + t) & p(a3 + t)) were supported by a model weight of 0.75 with no other competitive models (ΔAICc < 2; Table 1). Therefore, we continued to model the apparent survival with these two resighting-rate structure.

**Table 1:** Models used for assessment of age and time effects on resighting probabilities (p) of the Little Tern in Israel. ф structure for all models was ф (a4 –t/t/t/t).

	Model	AICc	ΔAICc	wAICc	np	Deviance
1	p(a4 + t)	1577.4	0	0.49159	28	126.6037
2	p(a3 + t)	1578.65	1.243	0.26405	29	125.6801
3	p(a3 - ././.)	1580.84	3.4338	0.0883	22	142.9102
4	p(a4 - ./././.)	1580.9	3.4917	0.08578	23	140.8376
5	p(a2 + t)	1585.84	8.4317	0.00726	26	139.3504
6	p(a2—t/t)	1589.52	12.1159	0.00115	31	132.2014
7	p(a3—t/t)	1590.82	13.4158	0.0006	36	122.5141
8	p(a2 - ./.)	1595	17.6002	0.00007	22	157.0766
9	p(a4—t/t)	1596.56	19.157	0.00003	39	121.5877
10	p(t)	1607.07	29.6619	0	26	160.5806
11	p(.)	1639.22	61.8147	0	18	209.7538

Models of resighting (*p*) probability for Little Terns, including Akaike's information criterion values (AICc), AICc differences (ΔAICc), and AICc Weight (wAICc), number of estimable parameters (np), and Deviance. Model Notation: '(.)' = constant over time; '(t)' = annual variation, '(T)' = linear time trend, a2–2 age classes (juvenile and adult), a4–4 age classes (juvenile, second year, third year and adult), (t/t) = including interaction between age and time, (+t) = additive effect of time (without interaction)

Apparent survival rates of juveniles decreased among the years of the study (Fig 1A) in four out of five of the top models (ΔAICc < 2; Table 2). The third model indicated constant survival of juveniles among the years but has less than a fifth of the weight compared to all the other top models. All of the top models indicated two age groups: in the first four models, first-year (juvenile), and after first year (adult) and in the fifth model, first and second year (as one age group) and adults. The survival of the adults did not vary across the years by the top two models. It did vary by year according to the third and the forth models but with less than half of the weight of the three other top models. Based on the most parsimonious model, the estimate of juvenile survival (first year) had decreased linearly across the years from 0.653 to 0.351, and was 0.493 ± 0.036 by the third model (with constant juvenile survival). The estimate of adult survival was 0.772 ± 0.029.

**Table 2:** Modelling of the influence effects on survival. Models used for assessment of age and time effects on apparent survival (ф) of the Little Tern (top 10 models; all the models are in supplementary S2 Table). Analysis used the two best p structures from the models of resighting probability.

	Model	AICc	ΔAICc	wAICc	np	Deviance
1	ф (a2—T/.) p(a3 + t)	1483.51	0.00	0.22	12	129.92
2	ф (a2—T/.) p(a4 + t)	1484.50	1.00	0.13	13	128.84
3	ф (a2 - ./t) p(a4 + t)	1485.01	1.51	0.10	15	125.18
4	ф (a2—T/T) p(a3 + t)	1485.28	1.78	0.09	13	129.62
5	ф (a2 - (a1 = a2)T/.) p(a3 + t)	1485.33	1.82	0.09	13	129.67
6	ф (a3—T/./.) p(a3 + t)	1485.57	2.07	0.08	13	129.91
7	ф (a2 - (a1 = a2)T/.) p(a4 + t)	1486.30	2.79	0.05	14	128.56
8	ф (a2—T/T) p(a4 + t)	1486.52	3.01	0.05	14	128.78
9	ф (a3—T/./.) p(a4 + t)	1486.54	3.03	0.05	14	128.80
10	ф (a3 - ./t) p(a3 + t)	1487.25	3.74	0.03	14	129.51

Models of annual survival (ф) for Little Terns. Model notation for ф '(.)' = constant over time; '(t)' = annual variation, '(T)' = linear time trend, a2–2 age classes (juvenile and adult), a3–3 age classes (juvenile, second year and adult), a4–4 age classes (t/t) = including interaction between age and time, (+t) = additive effect of time (without interaction)

## Discussion

In this study, we provide direct estimate of survival rates for adult and juvenile Little Terns, and test the hypothesis that survival rate increases with age among the younger age classes.

Survival rate is an important life-history feature that may shed light on the viability of a population [29]. We used data from extensive ringing (2011–2018) with 436 individuals ringed as juveniles, and resightings of more than 30% of the marked birds, in order to estimate directly adult and juvenile annual survival rate. The only other estimate of Little Tern adult survival was made by Tavecchia et al. (2005), where they reported an estimate of 0.9 [CI (0.836–0.963)]. However, their estimate included the survival probability and the probability of reaching or re-visiting a molting site; and, in addition, they considered as adults all birds older than 5 months (which are unlikely to have the same survival rate as older adults; [30]). Our estimate of 0.772 ± 0.029 adult annual survival rate is lower than the value found by Tavecchia et al. (2005), and also somewhat lower than a closely-related species (regarded as conspecifics), the Least Tern (*Sternula antillarum*), a New World tern species [31]. Studies of the Least Tern have reported annual survival rate estimates of 0.81–0.92 with 95% CI = 0.75–0.96 [32–34]. Our current finding provides evidence of a lower survival rate of first-year individuals. Most of our chosen models (ΔAICc < 2) showed a linear decrease in the juvenile survival estimates among the years of the study, which is surprising, mainly because we do not see a decrease in population size for those years. We do have some evidence of very low productivity for some of the years of the study, mainly as a result of disease and predation. It is important to note that the survival parameter includes the possibility of permanent emigration and therefore it is possible that the decline in the survival we observed represents an increase in the permanent emigration. In addition, we did have one model within the top five models (ΔAICc < 2) that indicated a constant survival rate among the years for the juveniles. The pattern of decrease in juvenile survival but not in population size suggests three main options. The first is that juvenile survival can vary among years [3], and it is therefore possible that juvenile survival in the better years overcomes the lower survival, such as that observed in Atlit. The second, is that the high number of breeding pairs in the colony is a novelty in the last few years (since 2012 –after the colony was fenced in), and therefore that although we do not yet see the effect of the low juvenile survival, we will witness a reduction in the number of breeding pairs in the coming years. The third possible explanation is that this population has arrived to its carrying capacity for this breeding colony. The number of breeding pairs has increased after the management efforts until reaching the highest number of breeding pairs recorded during the 1990s (and before the decrease trend had started). This can result in lower productivity of the colony and possibly in juveniles being progressively excluded from the breeding site and forced to emigrate (or die) [35,36].

In the case of a decline in a population, changes in juvenile survival can be seen while the adult survival rate will often remain constant despite the overall trend [37]. The differences in the pattern of survival between the two age groups can be due to several reasons: e.g., the conditions experienced during the early growth stages [38–43]; the differences in diet [44]; and the population density [45]. The resighting rate of juveniles was at least two-fold lower than that of adults. This corresponds to the fact that Little Terns usually do not breed until they are at least two-years-old, and, therefore, it is assumed that most of them remain in the African coastal water areas until they are ready to breed [3].

Our estimates of juvenile survival varied among the years (0.653 to 0.351) representing a linear decrease in survival. The value obtained by our third model (0.493 ± 0.036) which suggested constant survival for juvenile is within the estimate obtained by Tavecchia et al. (2005). The estimate reported by Tavecchia et al. (2005) was calculated by multiplying the value obtained for the survival between fledging and the pre-migratory stay at the molting site (June-August) and the value for adult survival (0.578 ± 0.160). In addition, the value we obtained was lower but close to estimates of the survival of Least Tern juveniles (0.56; [46]). We found no effect of adult age (after the first year) on survival. This may be because the survival rates of fledglings are much lower, while in the following few years survival rates remain relatively constant, as reported also for the Common Tern [47]. However, it is possible that our relatively lower sample size for each age class, which resulted in wide 95% confidence intervals for our survival rate estimates, prevented us from detecting small differences in survival rates among adult Little Terns. For example, studies on the Greater Flamingo (*Phoenicopterus ruber roseus*) found an increase in survival rate with age during the first few years [11,48].

The Little Tern populations have been declining in most of its breeding areas [14]. The main reasons for this pattern are habitat loss and deterioration [15]. In this study, we investigated the survival rates of juveniles and adults. Ongoing long-term research on Little Tern survival and reproduction will provide essential information to ensure the sustainability and protection of small and vulnerable populations throughout their distribution. Our survival estimates were somewhat lower compared to estimates for other, closely-related, species. Future long-term efforts in more colonies will allow a more precise modeling of age-specific curves, and more importantly, reveal whether the pattern of decrease in juvenile survival is shared with other populations in the world.

## Supporting information

S1 Table Description of a priori models used for analysis of apparent survival (ф) of the Little Tern. In the analysis we used the best p structure from the resighting probabilities models.
(DOCX)

S2 Table All models used for analysis of apparent survival (ф) of the Little Tern. In the analysis we used the two best p structures derived from the models of resighting probability.
(DOCX)
