# Supplementary material for: Age-dependent survival rate of the colonial Little Tern (Sternula albifrons)
Source: PLoS One. 2019 Dec 31;14(12):e0226819. doi: 10.1371/journal.pone.0226819 (PMC6938403; doi:10.1371/journal.pone.0226819)
Supplement: S1 Table — In the analysis we used the best p structure from the resighting probabilities models. (DOCX) [file pone.0226819.s001.docx]

**Table S1.** Description of a priori models used for analysis of apparent survival (ф) of the Little Tern. In the analysis we used the best p structure from the resighting probabilities models.

|  | **Model** | **Description of** Ф **sturcture** |
| --- | --- | --- |
| 1 | Ф (.) | Constant ф, no age or time effect |
| 2 | Ф(a2 - ./.) | Age effect, two age groups – juvenile and adult |
| 3 | Ф (a3 - ././.) | Age effect, three age groups – juvenile, second year and adult |
| 4 | Ф (a4 - ./././.) | Age effect, four age groups – juvenile, second year, third year and adult |
| 5 | Ф (a2 – (a1=a2)./.) | Age effect, two age groups – first and second year combine |
| 6 | Ф )t) | Annual time effect |
| 7 | Ф (t/t) | Annual time and age effect, two age groups |
| 8 | Ф (t/t/t) | Annual time and age effect, three age groups |
| 9 | Ф (t/t/t/t) | Annual time and age effect, four age groups |
| 10 | Ф (a2 – (a1=a2)t/t) | Annual time and age effect, two age groups – first and second year combine |
| 11 | Ф (t/.) | Annual time effect only on the juvenile, two age group, adult constant |
| 12 | Ф (t/./.) | Annual time effect only on the juvenile, three age groups |
| 13 | Ф (t/././.) | Annual time effect only on the juvenile, four age group |
| 14 | Ф (a2+t) | Two age groups with additive time effect |
| 15 | Ф (a2+t) | Three age groups with additive time effect |
| 16 | Ф (a2+t) | four age groups with additive time effect |
| 17 | Ф (a2 - T/.) | Two age groups with linear time effect on juvenile |
| 18 | Ф (a2 - T/T) | Two age groups with linear time effect on both juvenile and adult |
| 19 | Ф (a3 - T/./.) | Three age groups with linear time effect on juvenile |
| 20 | Ф (a4 - T/././.) | four age groups with linear time effect on juvenile |
| 21 | Ф (a2 – (a1=a2)T/.) | Two age groups, first and second year combine with linear time effect on the first age class. |
