# Supplementary material for: Age-dependent survival rate of the colonial Little Tern (Sternula albifrons)
Source: PLoS One. 2019 Dec 31;14(12):e0226819. doi: 10.1371/journal.pone.0226819 (PMC6938403; doi:10.1371/journal.pone.0226819)
Supplement: S2 Table — In the analysis we used the two best p structures derived from the models of resighting probability. (DOCX) [file pone.0226819.s002.docx]

**Table S2**. All models used for analysis of apparent survival (ф) of the Little Tern. In the analysis we used the two best p structures derived from the models of resighting probability.

|  | **Model** | **AICc** | **ΔAICc** | **wAICc** | **np** | **Deviance** |
| --- | --- | --- | --- | --- | --- | --- |
| 1 | ф (a2 - T/.) p(a3 + t) | 1483.51 | 0.00 | 0.22 | 12 | 129.92 |
| 2 | ф (a2 - T/.) p(a4 + t) | 1484.50 | 1.00 | 0.13 | 13 | 128.84 |
| 3 | ф (a2 - ./t) p(a4 + t) | 1485.01 | 1.51 | 0.10 | 15 | 125.18 |
| 4 | ф (a2 - T/T) p(a3 + t) | 1485.28 | 1.78 | 0.09 | 13 | 129.62 |
| 5 | ф (a2 - (a1=a2)T/.) p(a3 + t) | 1485.33 | 1.82 | 0.09 | 13 | 129.67 |
| 6 | ф (a3 - T/./.) p(a3 + t) | 1485.57 | 2.07 | 0.08 | 13 | 129.91 |
| 7 | ф (a2 - (a1=a2)T/.) p(a4 + t) | 1486.30 | 2.79 | 0.05 | 14 | 128.56 |
| 8 | ф (a2 - T/T) p(a4 + t) | 1486.52 | 3.01 | 0.05 | 14 | 128.78 |
| 9 | ф (a3 - T/./.) p(a4 + t) | 1486.54 | 3.03 | 0.05 | 14 | 128.80 |
| 10 | ф (a3 - ./t) p(a3 + t) | 1487.25 | 3.74 | 0.03 | 14 | 129.51 |
| 11 | ф (a2 - ./.) p(a4 + t) | 1488.17 | 4.66 | 0.02 | 12 | 134.58 |
| 12 | ф (a2 - ./.) p(a3 + t) | 1488.36 | 4.85 | 0.02 | 11 | 136.85 |
| 13 | ф (a3 + t) p(a3 + t) | 1488.79 | 5.29 | 0.02 | 17 | 124.77 |
| 14 | ф (a2 + t) p(a4 + t) | 1489.77 | 6.26 | 0.01 | 17 | 125.74 |
| 15 | ф (a3 - ././.) p(a4 + t) | 1490.11 | 6.61 | 0.01 | 13 | 134.45 |
| 16 | ф (a3 - ././.) p(a3 + t) | 1490.41 | 6.90 | 0.01 | 12 | 136.82 |
| 17 | ф (a2 + t) p(a3 + t) | 1491.15 | 7.64 | 0.00 | 16 | 129.22 |
| 18 | ф (a2 - t/.) p(a3 + t) | 1491.34 | 7.83 | 0.00 | 16 | 129.42 |
| 19 | ф (a2 - t/.) p(a4 + t) | 1492.33 | 8.82 | 0.00 | 17 | 128.30 |
| 20 | ф (a4 + t) p(a4 + t) | 1492.55 | 9.04 | 0.00 | 19 | 124.31 |
| 21 | ф (a4 + t) p(a3 + t) | 1493.10 | 9.59 | 0.00 | 18 | 126.97 |
| 22 | ф (a3 - t/./.) p(a3 + t) | 1493.43 | 9.93 | 0.00 | 17 | 129.41 |
| 23 | ф (a2 - t/ t) p(a4 + t) | 1493.52 | 10.02 | 0.00 | 20 | 123.16 |
| 24 | ф (a3 - t/./.) p(a4 + t) | 1494.39 | 10.89 | 0.00 | 18 | 128.27 |
| 25 | ф (a4 - T/././.) p(a4 + t) | 1495.62 | 12.11 | 0.00 | 15 | 135.79 |
| 26 | ф (a2 - t/t) p(a3 + t) | 1496.82 | 13.31 | 0.00 | 20 | 126.46 |
| 27 | ф (a2 - (a1=a2)t/t) p(a3 + t) | 1498.63 | 15.12 | 0.00 | 16 | 136.71 |
| 28 | ф (a3 + t) p(a4 + t) | 1499.17 | 15.66 | 0.00 | 18 | 133.04 |
| 29 | ф (a2 - (a1=a2)t/t) p(a4 + t) | 1502.33 | 18.82 | 0.00 | 18 | 136.20 |
| 30 | ф (a4 - t/t/t/t) p(a3 + t) | 1502.67 | 19.16 | 0.00 | 26 | 119.47 |
| 31 | ф (a4 - ./././.) p(a4 + t) | 1502.98 | 19.47 | 0.00 | 14 | 145.24 |
| 32 | ф (a4 - t/././.) p(a4 + t) | 1503.47 | 19.96 | 0.00 | 19 | 135.22 |
| 33 | ф (a4 - t/t/t/t) p(a4 + t) | 1504.80 | 21.30 | 0.00 | 28 | 117.27 |
| 34 | ф (t) p(a4 + t) | 1506.93 | 23.42 | 0.00 | 16 | 145.00 |
| 35 | ф (.) p(a4 + t) | 1508.77 | 25.26 | 0.00 | 11 | 157.25 |
| 36 | ф (a2 - (a1=a2)./.) p(a4 + t) | 1509.57 | 26.07 | 0.00 | 12 | 155.99 |
| 37 | ф (.) p(a3 + t) | 1510.74 | 27.24 | 0.00 | 10 | 161.29 |
| 38 | ф (a4 - T/././.) p(a3 + t) | 1510.86 | 27.36 | 0.00 | 14 | 153.12 |
| 39 | ф (a2 - (a1=a2)./.) p(a3 + t) | 1510.95 | 27.45 | 0.00 | 11 | 159.44 |
| 40 | ф (a3 - t/t/t) p(a4 + t) | 1512.50 | 29.00 | 0.00 | 25 | 131.46 |
| 41 | ф (t) p(a3 + t) | 1513.22 | 29.71 | 0.00 | 15 | 153.39 |
| 42 | ф (a4 - ./././.) p(a3 + t) | 1520.31 | 36.80 | 0.00 | 13 | 164.65 |
